# Pentylenetetrazol-Induced Epileptiform Activity Affects Basal Synaptic Transmission and Short-Term Plasticity in Monosynaptic Connections

**DOI:** 10.1371/journal.pone.0056968

**Published:** 2013-02-20

**Authors:** Carlo Natale Giuseppe Giachello, Federica Premoselli, Pier Giorgio Montarolo, Mirella Ghirardi

**Affiliations:** 1 Section of Physiology, Department of Neuroscience, University of Turin, Turin, Italy; 2 Istituto Nazionale di Neuroscienze, Turin, Italy; Centre national de la recherche scientifique, University of Bordeaux, France

## Abstract

Epileptic activity is generally induced in experimental models by local application of epileptogenic drugs, including pentylenetetrazol (PTZ), widely used on both vertebrate and invertebrate neurons. Despite the high prevalence of this neurological disorder and the extensive research on it, the cellular and molecular mechanisms underlying epileptogenesis still remain unclear. In this work, we examined PTZ-induced neuronal changes in *Helix* monosynaptic circuits formed *in vitro*, as a simpler experimental model to investigate the effects of epileptiform activity on both basal release and post-tetanic potentiation (PTP), a form of short-term plasticity. We observed a significant enhancement of basal synaptic strength, with kinetics resembling those of previously described use-dependent forms of plasticity, determined by changes in estimated quantal parameters, such as the readily releasable pool and the release probability. Moreover, these neurons exhibited a strong reduction in PTP expression and in its decay time constant, suggesting an impairment in the dynamic reorganization of synaptic vesicle pools following prolonged stimulation of synaptic transmission. In order to explain this imbalance, we determined whether epileptic activity is related to the phosphorylation level of synapsin, which is known to modulate synaptic plasticity. Using western blot and immunocytochemical staining we found a PTZ-dependent increase in synapsin phosphorylation at both PKA/CaMKI/IV and MAPK/Erk sites, both of which are important for modulating synaptic plasticity. Taken together, our findings suggest that prolonged epileptiform activity leads to an increase in the synapsin phosphorylation status, thereby contributing to an alteration of synaptic strength in both basal condition and tetanus-induced potentiation.

## Introduction

Epilepsy, a neurological disorder affecting 0.5–1% of the population, is characterised by abnormal electrical discharges in the brain and seizures. Despite high prevalence and extensive research, the mechanisms underlying both the onset and the progression of epilepsy still remain unclear.

In parallel to vertebrates, invertebrate preparations have been valuable tools in the study of epileptogenesis. A large body of literature uses the buccal ganglia of the land snail, genus *Helix*, as a good experimental system to study epileptiform activity [1]. *Helix* neurons are sensitive to epileptogenic drugs and have been extensively investigated as regards their electrophysiological [2]–[4], morphological [5] and epileptological [6]–[8] properties. Generally, epileptiform activity is often induced by local application of epileptogenic drugs, such as pentylenetretazol (PTZ), commonly employed in both vertebrate and invertebrate preparations. When PTZ is applied to *Helix* neurons, the response discharge pattern changes rapidly from a pre-convulsive state to paroxysmal depolarisation shift (PDS) in the convulsive phase [9]. PDS is an abnormal prolonged depolarisation with repetitive spiking associated with alterations in spike generation and is followed by a rapid repolarisation and hyperpolarisation [10]–[12]. The results obtained from experiments on intact ganglia performed to date are difficult to analyse, due to complexity of the neuronal networks. Moreover, inhomogeneous delivery of the topically applied drug results in an inconsistent mix of direct and indirect effects. Therefore, it is beneficial to have a simpler experimental model to investigate the molecular mechanisms underlying epileptic-like activity. Invertebrate cell cultures are particularly advantageous for several reasons (reviewed in [13]): first, identifiable *Helix* neurons can be isolated from their synaptic inputs and monosynaptic connections can be formed *in vitro* [14]; secondly, PDSs evoked by PTZ administration in snail neurons resemble those in mammalian cortical neurons [3], [9].

Several lines of evidence indicate that synapsin deletion or mutation is associated with epileptic behaviour [15]–[21]. Synapsins are a family of vesicle-associated phospho-proteins clearly implicated in the modulation of neurotransmitter release by controlling the availability of synaptic vesicles for exocytosis [22]. These proteins reversibly associate with synaptic vesicles, actin, microtubules, and other synapsin isoforms in a phosphorylation-dependent manner [23]–[27]. In presynaptic terminals, synaptic vesicles are organized in distinct pools that are functionally defined depending on their availability for release [28]–[30]. The reserve pool (RP) is defined as a depot of synaptic vesicles from which release is only triggered during intense stimulation [31]–[34]. The readily releasable pool (RRP) is a population of neurotransmitter quanta that are available for immediate release [35]–[37]. It has been proposed that synapsin, through its phosphorylation, plays an important role in both the maintenance of the RP [15], [16], [28], and the mobilisation of vesicles from the RP to the RRP under conditions of increased presynaptic activity [38]–[42]. Therefore, abnormal synapsin function may induce alterations in synaptic neurotransmission and its modulation that could also lead to an epileptic phenotype.

In this work, we take advantage of *in vitro* monosynaptic connections between B2 neurons isolated from the *Helix* buccal ganglia to investigate the effect of PTZ-induced epileptiform activity on basal synaptic transmission and post-tetanic potentiation (PTP), a form of short-term plasticity. Since we previously demonstrated that PTP expression is directly correlated to the phosphorylation of synapsin domain A and B [43], [44], we also evaluated the involvement of these phospho-sites in PTZ-induced plastic phenomena.

## Results

### PTZ application induces epileptiform activity in *Helix* cocultures

**Figure 1:**
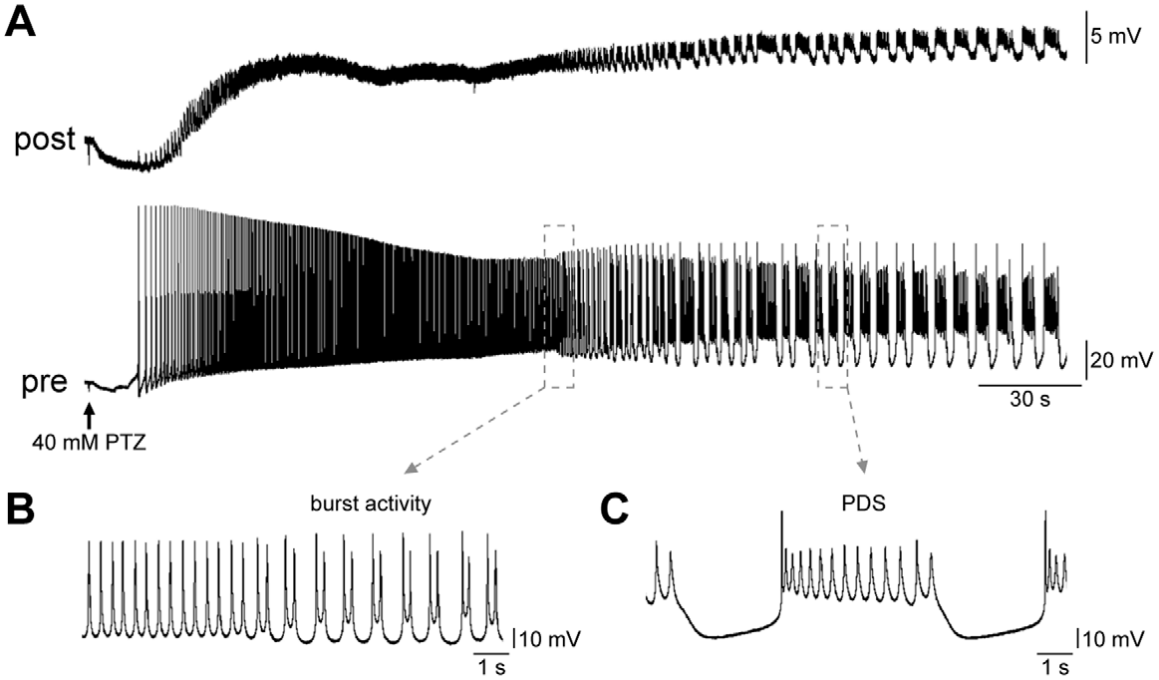
PTZ application induces epileptiform activity in *Helix* B2 –**B2 monosynaptic connections.** (A) Representative electrophysiological recording of PTZ-induced epileptiform activity from a B2–B2 connection in culture. EPSPs were recorded from the postsynaptic cell kept hyperpolarised to 30 mV below its membrane potential to prevent firing. The application of 40 mM PTZ rapidly generated sustained firing activity in the presynaptic neuron. Afterwards, the discharge pattern changed from single spikes to a burst firing pattern (B) and PDS episodes (C). PDS is an abnormal prolonged depolarisation step with superimposed action potentials.

The properties of *Helix* B2 neurons have been extensively described in both *in vivo* and *in vitro* conditions [4], [45], [46]. When paired in culture, these neurons reliably form excitatory synaptic connections, and display several forms of activity-dependent synaptic enhancement, including PTP [44]. Both synaptically isolated and chemically interconnected B2 neurons are usually silent in culture [47]. Soon after the application of PTZ, we observed in these cells a peculiar neural activity that can be divided into three phases (Fig. 1A): (1) a rapid depolarisation that triggers action potential firing; (2) a progressive acceleration of firing activity; (3) an epileptic-like phase, in which the discharge pattern rapidly changed to a regular bursting-rhythm composed of irregular spikes (Fig. 1B) and, occasionally, PDS (Fig. 1C). In these cells PTZ treatment induces discharge patterns similar to a PDS described in the mammalian brain [3], in an extremely dose-dependent fashion. Previous studies on intact buccal ganglia defined 10–20 mM PTZ as subthreshold concentrations, while 40 mM PTZ (epileptogenic dose) has been extensively used to generate epileptiform activity in intact buccal ganglia [9], [48]. In order to determine the appropriate dose in culture, we firstly investigated the effect of PTZ on synaptically isolated B2 neurons, perfused with varying concentrations. We observed that cells treated with final concentrations of 20 mM and 40 mM showed a similar increase in discharge pattern reaching a mean firing rate of 1.04±0.22 spikes/s (*n* = 12) and 1.12±0.14 spikes/s (*n* = 15), respectively. However, while a dose of 20 mM PTZ elicited a long-lasting regular-spiking, the majority of 40 mM PTZ-treated cells (i.e. >80%) changed their firing pattern to paroxysmal activity within a few minutes of drug application. Thus, we consider 40 mM PTZ as a useful concentration to induce epileptiform activity in our experiments. After washout, we verified that exposure to PTZ did not induce any alteration in membrane potential, or input resistance, in B2 neurons hyperpolarized to about -80 mV, 30 mV negative to their resting potential (data not shown).

**Figure 2:**
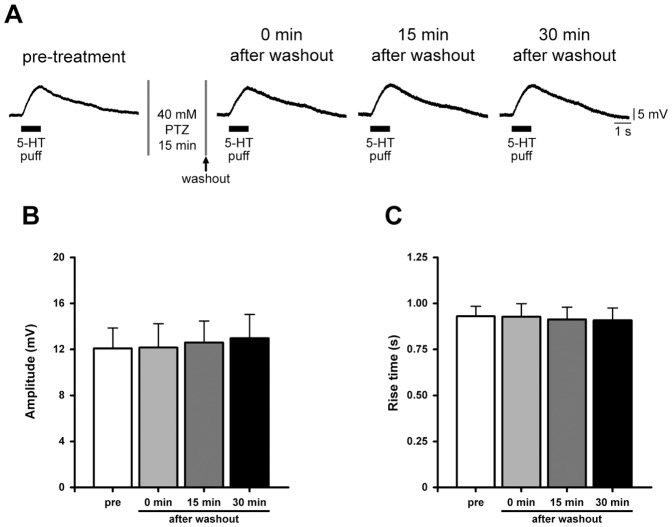
PTZ application does not compromise B2 postsynaptic response to locally applied neurotransmitter. (A) Sample electrophysiological recordings showing the response to locally applied 20 µM serotonin pulses (5-HT for 1 s; 10 psi) before and after the exposure to PTZ (at 0, 15 and 30 minutes after drug washout). (B) Bar graph of the mean depolarization evoked by neurotransmitter application. No detectable changes were observed among the examined groups. (C) Bar graph of the corresponding rise times.

In order to check possible alterations in postsynaptic receptor functionality, we evaluated the response to neurotransmitter by testing the overall sensitivity of B2 neurons to local serotonin application with a glass electrode attached to a pneumatic picopump (Fig. 2A,B). Fixed volumes of a 20 µM serotonin solution were delivered close to neurons, each time placing the tip of the electrode at the same distance from the soma, i.e. 50 µm, corresponding to a representative B2 cell diameter. We found that focal neurotransmitter application induced a similar membrane depolarisation before (12.09±1.77 mV, *n* = 6) and soon after PTZ washout (12.16±2.06 mV). Comparable responses were observed testing the same cells both 15 and 30 minutes after drug removal, 12.61±1.87 mV and 12.98±2.05 mV, respectively. No statistically significant changes were found in the corresponding rise times (pre-treatment: 0.93±0.05 s; 0 min after washout: 0.93±0.07 s; 15 min after washout: 0.91±0.06 s; 30 min after washout: 0.91±0.07 s; *n* = 6; Fig. 2C).

### PTZ-induced epileptiform activity alters basal synaptic transmission

Several factors including changes in synaptic transmission and its modulation may participate in the induction and the maintenance of seizure activity. To better investigate whether epileptic-like activity affects excitatory transmission, we recorded monosynaptic B2–B2 connections in culture. During PTZ exposure, we generally observed a rapid potentiation of synaptic strength followed by a progressive decline in amplitude of EPSPs recorded from the postsynaptic cell. This resulting reduction in postsynaptic response may be ascribed to synaptic fatigue, that is an attenuation of synaptic efficacy during excessive use, such as during an epileptic seizure. Considering these observations, we evaluated changes in basal synaptic transmission that occurred immediately, or resulted from a late rebound of epileptiform activity.

**Figure 3:**
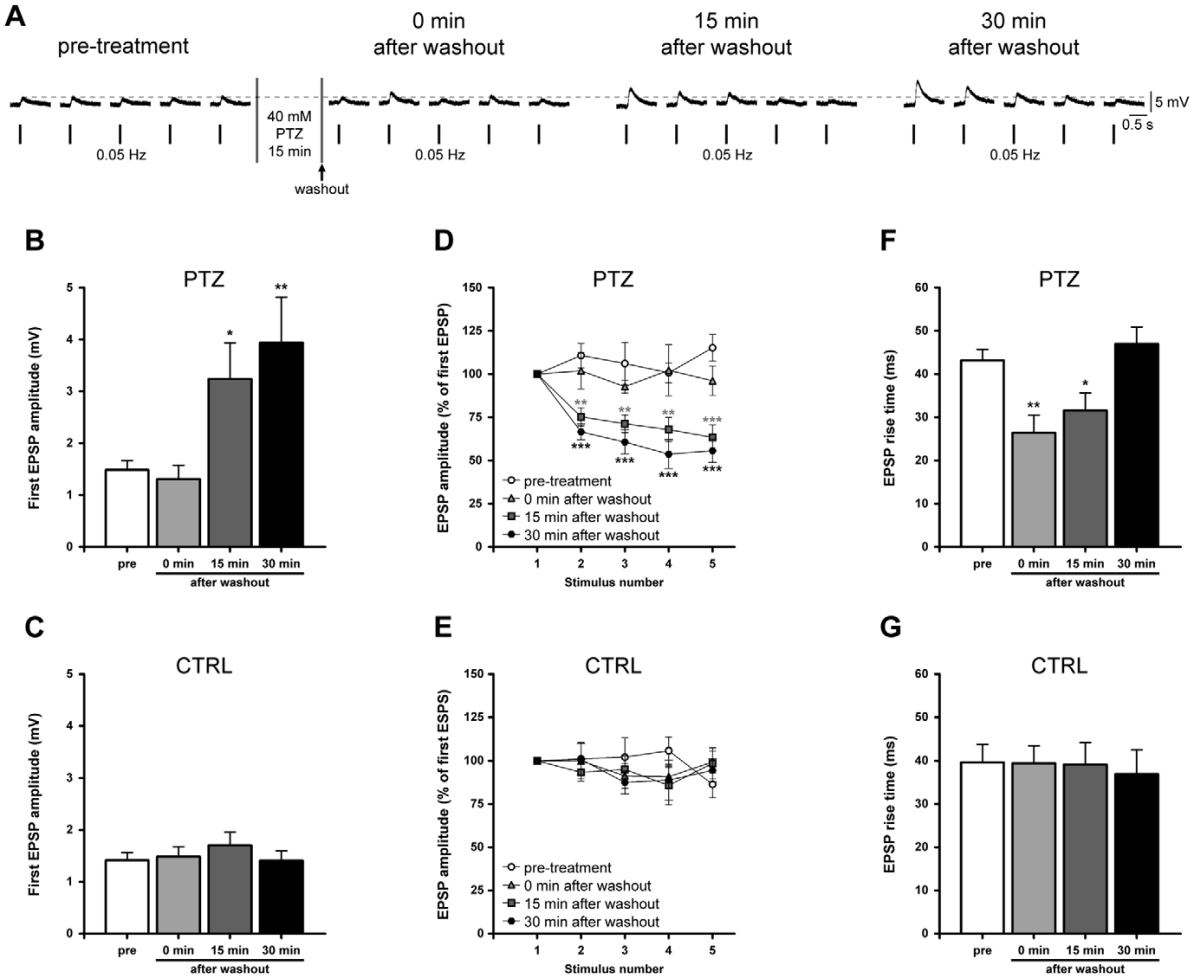
Epileptiform activity induces a use-dependent synaptic potentiation at B2 –**B2 synapses.** (A) Sample electrophysiological recordings of five consecutive EPSPs evoked at the basal rate of 0.05 Hz. Synapses were tested before and every 15 minutes after PTZ treatment and washout. An enhancement of synaptic strength was detectable at both 15 and 30 minutes after drug washout. (B) Bar graph of the mean amplitude of the first EPSP in treated synapses. EPSP amplitudes recorded before and at the end of PTZ exposure were similar. Conversely, a significant potentiation of 218.30±46.77% and 265.50±59.31% of pre-treatment value occurred at 15 and 30 minutes post-treatment, respectively. (C) Unchanged values were measured in the control group at any time-point. (D,E) Time course of EPSP amplitude in treated and untreated monosynaptic connections stimulated at 0.05 Hz. Values were expressed as percentages of the first EPSP amplitude to better appreciate the trend of EPSPs recorded at 15 and 30 minutes after PTZ washout. (F) Bar graph of EPSP rise times measured at each experimental time-point, showing that epileptiform activity temporarily speeded up the kinetics of release. (G) No significant difference was found in control group.

Five consecutive EPSPs, evoked at the frequency of 0.05 Hz, were recorded from monosynaptic B2–B2 synapses before (pre-treatment) and after (0 min after washout) the perfusion of a 40 mM PTZ solution for 15 minutes. We also determined the reversibility of PTZ action by testing the same synapses 15 and 30 minutes after drug washout (Fig. 3A). We observed significant changes in amplitude of the first evoked EPSP (*n* = 13, F_(3,48)_ = 6.42, *P* = 0.001, one-way ANOVA, Fig. 3B). Values recorded before and at the end of PTZ application were similar, 1.48±0.18 mV and 1.31±0.27 mV, respectively. Conversely, a statistically significant enhancement occurred 15 and 30 minutes after washout, reaching 218.30±46.77% (15 min after washout: 3.24±0.70 mV) and 265.50±59.31% (30 min after washout: 3.94±0.88 mV) of pre-treatment value. In the control group (no PTZ application), no difference in the mean amplitude of the first EPSP was detected at each time-point (pre-treatment: 1.41±0.15 mV; 0 min after washout: 1.48±0.19 mV; 15 min after washout: 1.71±0.25 mV; 30 min after washout: 1.41±0.19 mV; *n* = 6, Fig. 3C).

At 15 and 30 minutes post-treatment, in addition to the enhancement in amplitude of the first EPSP, we observed an exponential decrease in amplitude of the following EPSPs (from second to fifth) during basal stimulation with a time constant of about 20–30 s (*n* = 13, Fig. 3D). A two-way ANOVA for repeated measures confirmed an overall significant effect of treatment (F_(3,48)_ = 12.72, *P*<0.0001) and stimulus number (F_(4,48)_ = 6.00, *P* = 0.0001) and a significant treatment by stimulus interaction (F_(12,192)_ = 3.03, *P*<0.001). Considering the amplitudes of the evoked EPSPs, we found that the fifth EPSP was significantly decreased in its amplitude to 63.43±7.21% (15 min after washout) and 55.70±6.76% (30 min after washout) with respect to the first EPSP. In both cases, synaptic strength depotentiated to a plateau value of 1.44±0.98 mV at 151minutes, and 1.89±0.59 mV at 30 minutes after washout. These amplitudes were similar to mean basal EPSP amplitudes recorded before (1.65±0.20 mV) and soon after the treatment (1.44±0.26 mV) where no depression was detected. In the control group, no significant variation in the EPSP amplitude was observed at the different time-points (mean basal EPSP amplitude: pre-treatment: 1.40±0.16 mV; 0 min after washout: 1.43±0.20 mV; 15 min after washout: 1.56±0.19 mV; 30 min after washout: 1.45±0.13 mV; *n* = 6; Fig. 3E). In both groups, none of the recorded neurons displayed spontaneous activity in the time window between stimulations, confirming that *Helix* neurons are generally silent in culture.

These results may indicate the presence of a novel form of synaptic potentiation, as a late rebound of epileptiform activity, which is “use”- but not time-dependent, whereby the increased strength is similar at 15 and 30 minutes following PTZ washout. This enhancement was depotentiated following two or three action potentials.

Finally, we measured the EPSP rise times at all time-points of treated synapses and we found that PTZ consistently speeded up the kinetics of release (*n* = 13, F_(3,48)_ = 6.48, *P*<0.001, one-way ANOVA, Fig. 3F). Based on recordings of B2-B2 synapses, significantly shortened EPSP rise times were present at time 0 and 15 minutes post-treatment, 26.39±4.11 ms (*P*<0.01, Bonferroni’s *post hoc* test) and 31.58±4.06 ms (*P*<0.05, Bonferroni’s *post hoc* test), respectively, compared to pre-treatment values (43.14±2.58 ms). Subsequently, at 30 minutes after washout (47.01±3.91 ms), the value returned to a similar level as in the pre-treatment group. No such alteration was observed in the control group, where rise times were not statistically different at any time-point (pre-treatment: 39.64±4.10 ms; 0 min after washout: 39.44±3.99 ms; 15 min after washout: 39.12±4.99 ms; 30 min after washout: 36.90±5.67 ms; *n* = 6; Fig. 3G).

### PTZ-induced epileptiform activity alters the readily releasable pool and the release probability

**Figure 4:**
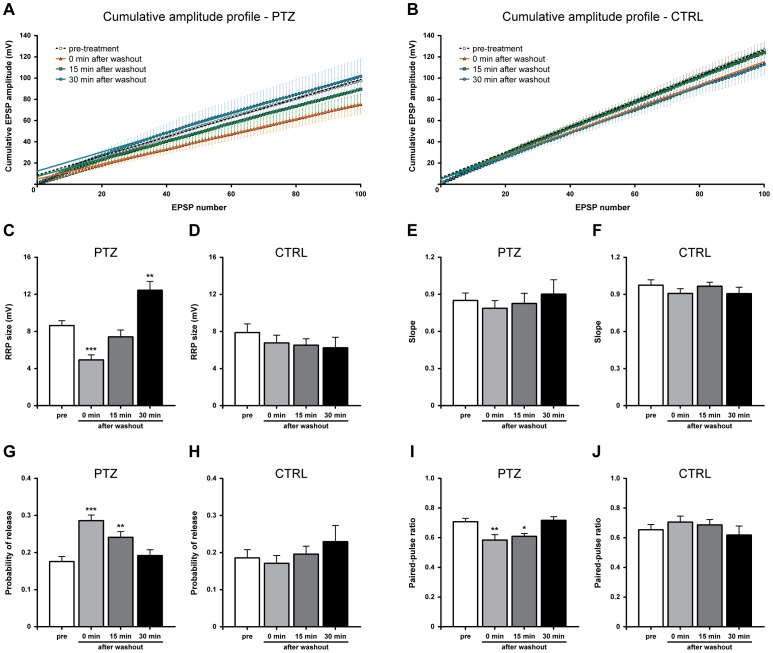
Epileptiform activity induces opposite changes in the RRP and P_rel._ (A,B) Cumulative curves of EPSP amplitudes evoked at 10 Hz at different time-points. The linear part was fitted as described in Methods. (C) Following PTZ exposure, the estimated RRP size, expressed in mV, significantly decreased soon after washout, recovered at 15 minutes and then increased 30 minutes post-treatment. (D) On the contrary, the RRP remained unchanged in control group. (E,F) Bar graph of mean values of slopes measured from the linear regression lines, indicating similar vesicle replenishment rates in both treated and untreated synapses. (G) Bar graph of P_rel_ showing an increase in this parameter following epileptiform activity which returns to pre-treatment values at 30 minutes after drug washout. (H) No alteration was measured in untreated synapses. (I) Histogram of mean PPR values obtained from treated B2–B2 synapses. PTZ exposure transiently reduced PPR with a complete recovery at 30 minutes after washout. (J) No detectable changes in PPR were observed in control group.

Since altered synaptic transmission may result from changes in quantal parameters of release, namely the size of the RRP and the release probability (P_rel_), we characterised synaptic dynamics with a rapid depletion protocol evoking EPSPs at 10 Hz (100 stimuli) in both PTZ-treated and untreated monosynaptic connections at different time-points (Fig. 4A,B). Data were analysed by plotting cumulative EPSPs and fitting a regression line through the linear portion of the plot which represents a balance between the rate of vesicle release and the continual replenishment of the RRP. Generally, we found that EPSPs declined to a low steady-state amplitude by the twenty-fifth action potential; therefore values in the range of 25–100 stimuli were included in this analysis in order to obtain the best linear fitting (all R^2^ values were >0.99). From cumulative amplitude profiles, the *y* intercept value back-extrapolated from the regression line yields a rough estimation of the RRP size at the start of the high frequency stimulation, while the slope provides an indicator of the RRP replenishment rate [49]–[50].

We found that the estimated size of the RRP was significantly affected by PTZ-induced epileptiform activity (*n* = 13, F_(3,48)_ = 19.30, *P*<0.0001, one-way ANOVA; Fig. 4C). The mean RRP size decreased from 8.64±0.52 mV (pre-treatment) to 4.93±0.54 mV, measured soon after PTZ washout (*P*<0.001, Bonferroni’s *post hoc* test), but completely recovered 15 minutes later (7.41±0.75 mV). Afterwards, we observed a statistically significant increase in the RRP size at 30 minutes post-treatment, (12.44±0.96 mV; *P*<0.01, Bonferroni’s *post hoc* test). Conversely, mock-treated synapses in the control group showed similar values at different time-points (pre-treatment: 7.88±0.93 mV; 0 min after washout: 6.77±0.83 mV; 15 min after washout: 6.52±0.70 mV; 30 min after washout: 6.24±1.14 mV; *n* = 5, Fig. 4D).

No statistically significant changes in slope value were found in untreated synapses (pre-treatment: 0.97±0.04; 0 min after washout: 0.91±0.04; 15 min after washout: 0.97±0.03; 30 min after washout: 0.91±0.05; *n* = 5; Fig. 4E), as well as PTZ-treated cells (pre-treatment: 0.85±0.06; 0 min after washout: 0.79±0.06; 15 min after washout: 0.82±0.08; 30 min after washout: 0.90±0.12; *n* = 13; Fig. 4F), suggesting that the exposure to this epileptogenic drug did not affect the replenishment rate of the RRP.

The probability of release (P_rel_) was calculated as the ratio between the first EPSP in the train and the estimated RRP. Indeed, P_rel_ exhibited a reversible increase following epileptiform activity (*n* = 13, F_(3,48)_ = 11.59, *P*<0.0001, one-way ANOVA; Fig. 4G) from 0.17±0.01 to 0.29±0.02 (*P*<0.001, Bonferroni’s *post hoc* test), measured before and after PTZ treatment, respectively. During recovery, this parameter slowly decayed to 0.24±0.02 at 15 minutes after washout (*P*<0.01, Bonferroni’s *post hoc* test), and reached the pre-treatment value only 30 minutes later (0.19±0.02). On the other hand, no statistically significant difference was found in the control group (pre-treatment: 0.18±0.02; 0 min after washout: 0.17±0.02; 15 min after washout: 0.19±0.02; 30 min after washout: 0.23±0.04; *n* = 5, Fig. 4H).

As a second approach, we also investigated changes in P_rel_ by measuring the paired-pulse ratio (PPR) from each train. PPR is defined as the ratio between the amplitude of the second EPSP and that of the first. One-way ANOVA analysis revealed a significant effect of PTZ exposure (*n* = 13, F_(3,48)_ = 6.531, *P*<0.001; Fig. 4I), with a decrease in PPR value from 0.71±0.02 (pre-treatment) to 0.58±0.04 (*P*<0.01, Bonferroni’s *post hoc* test) after PTZ washout (0 min after washout) and 0.61±0.02 (*P*<0.05, Bonferroni’s *post hoc* test) at 15 minutes after drug removal. In agreement with previous results, we detected a complete recovery of PPR at 30 minutes after PTZ washout (0.72±0.02). Since previous studies have shown that PPR is inversely related to P_rel_ [51]–[53], such changes may indicate a transient increase in P_rel_ following PTZ treatment. Considering the control group, we observed similar values at all time-points (pre-treatment: 0.65±0.03; 0 min after washout: 0.71±0.04; 15 min after washout: 0.69±0.04; 30 min after washout: 0.62±0.06; *n* = 5; Fig. 4J).

Based on this analysis, we conclude that epileptiform activity not only temporarily increases the probability of release, but also induces significant changes in the RRP size at different time-points. Imbalances of these two parameters may explain the altered synaptic strength observed during basal transmission.

### PTZ treatment impairs post-tetanic potentiation

**Figure 5:**
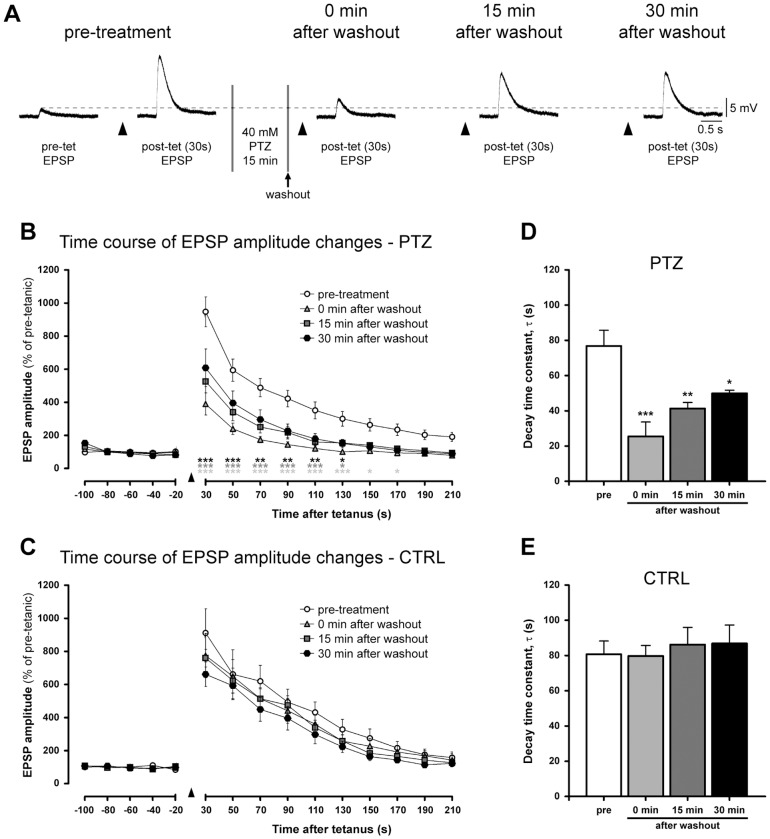
Epileptiform activity impairs PTP induction and decay. (A) Sample electrophysiological recordings of the first post-tetanic EPSPs evoked 30 seconds after the tetanic stimulation (arrowhead) at B2–B2 synapses. Dashed line indicates the amplitude of the pre-treatment EPSP evoked before tetanisation. PTP was tested before (pre-treatment) and after 40 mM PTZ treatment at different time-points (0, 15 and 30 minutes after washout). (B,C) The time course of EPSP amplitude changes in PTZ-treated and control synapses. Values are normalized to the average amplitude of the last five pre-tetanic EPSPs. A strong impairment in PTP amplitude and decay was detected only in the treated group. (D) Bar graph of the PTP decay time constants measured in treated B2–B2 synapses showing a large reduction in τ. (E) Conversely, values in control group were similar at all time-points.

Besides basal synaptic transmission, we also investigated whether chemically-induced epileptiform activity affected activity-dependent synaptic enhancement, i.e. PTP whose properties have been previously described in B2-B2 monosynaptic connections [44]. To this aim, we induced PTP before and after perfusion of 40 mM PTZ (*n* = 15; Fig. 5A,B) or vehicle alone (*n* = 5; Fig. 5C) as a control, then we determined the reversibility of PTZ effect in the same synapses by testing PTP at 15 and 30 minutes after drug washout. We observed that PTZ treatment strongly compromised the expression of PTP, which was not restored even after 30 minutes after drug removal. A two-way ANOVA for repeated measures confirmed a significant effect of treatment (F_(3,56)_ = 11.58, *P*<0.0001) and time (F_(14,56)_ = 113.4, *P*<0.0001) and a significant treatment by time interaction (F_(42,784)_ = 7.61, *P*<0.0001).

We also analysed the time course of PTP decay fitted to a mono-exponential function to estimate the decay constant (τ) in both treated and untreated synapses. We measured a τ value of 76.78±8.95 s in the pre-treatment group, 25.50±8.15 s soon after the washout, 41.35±3.37 s at 15 minutes after washout and 49.96±1.71 s at 30 minutes (Fig. 5D). On the whole, all the experimental points recorded after PTZ treatment showed a significant reduction in PTP decay time (F_(3,56)_ = 10.67, *P*<0.0001, one-way ANOVA). The τ values calculated in control group did not significantly change during the stimulation protocol (Fig. 5E; pre-treatment: 80.70±7.57 s; 0 min after washout: 79.77±5.91 s; 15 min after washout: 86.21±9.76 s; 30 min after washout: 86.84±10.45 s).

Taken together, these results indicate that epileptiform activity induced by PTZ application compromises the capacity of B2–B2 excitatory synapses to undergo activity-dependent synaptic plasticity, affecting PTP amplitude and its decay. Therefore, we can infer that PTZ treatment affects not only the RRP but also the RP, preventing synaptic potentiation.

### PTZ treatment induces the phosphorylation of synapsin domain A and B

Since we observed that PTZ treatment affected both the RRP and the RP, possibly altering the reorganisation of these synaptic vesicle pools, we tested the hypothesis that this action might be mediated by synapsin phosphorylation. *Helix* synapsin (helSyn) contains well-conserved sites for PKA and CaMKI/IV in domain A, named site 1, and for MAPK/Erk in domain B, named sites 4 and 5. We have previously published research on the role of both sites in the expression of PTP and formation of functional connections [43], [44]. Consequently we focused our attention on these sites to evaluate changes in their phosphorylation status induced by epileptic-like activity. As a first approach, we performed a western blot analysis to investigate PTZ effects on isolated ganglia, compared with those induced by vehicle alone (negative control) or with high-extracellular KCl application, commonly used to induce depolarisation (positive control). Endogenous synapsin, both total and phosphorylated, was detected with custom-designed rabbit polyclonal antibodies. The specificity of α-P_1_helSyn and α-P_4,5_helSyn was tested by means of a dephosphorylation assay on blotted proteins (see Methods). No signal was detectable, not even after a prolonged exposure, whereas bands were clearly present in not-dephosphorylated blots or after reprobing with α-helSyn antibody, thus verifying the reliability of our antibodies (data not shown).

**Figure 6:**
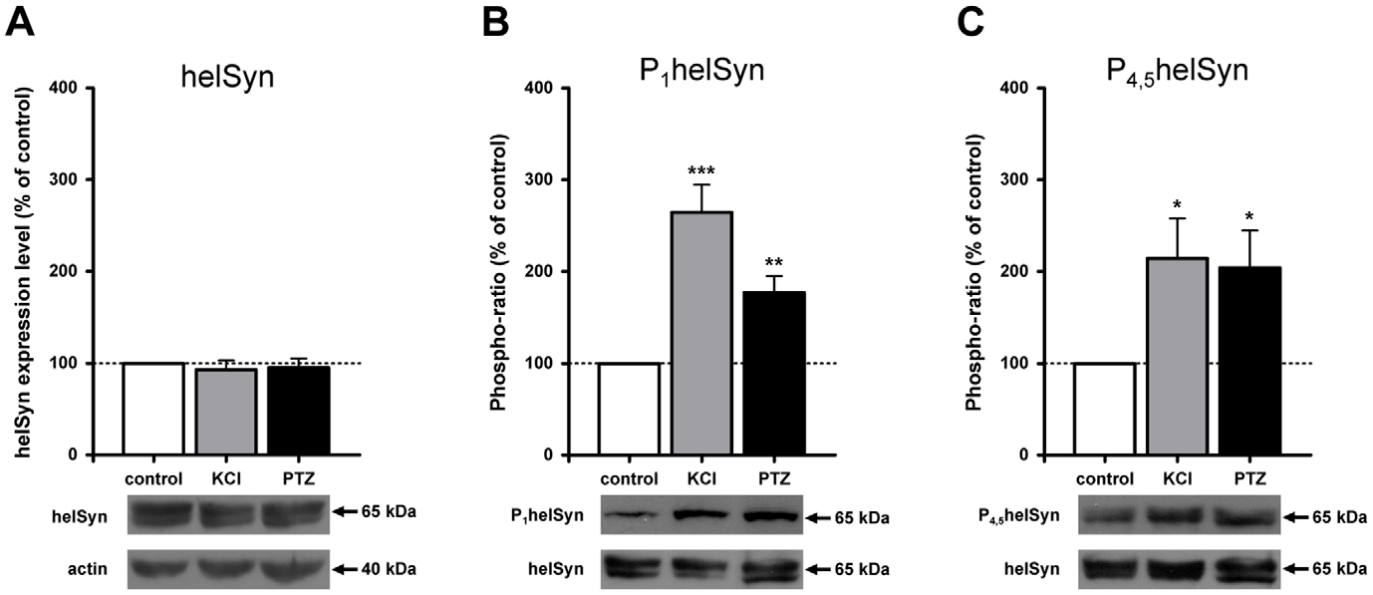
PTZ treatment increases synapsin phosphorylation of both domain A and B in *Helix* ganglia. (A) Western blot analysis of helSyn expression level normalized to actin along with histograms presenting average densitometric values. Values were similar in all experimental groups. (B,C) Mean density value of phospho-helSyn compared to that of total helSyn (phospho-ratio). Both phospho-site levels were increased by KCl or PTZ application.

We checked the effect of PTZ on the total level of synapsin (Fig. 6A) by calculating the ratio of volume (intensity*mm^2^), adjusted for the background, between helSyn and actin bands. No significant differences were found between PTZ- or KCl-treated samples and control, thus indicating that the synapsin total level remained unchanged after treatments. According to these results, we decided to express the level of phosphorylation as a ratio between phosphorylated and total synapsin immunoreactivity (defined as phospho-ratio). To this aim, immunoblots hybridised with α-P_1_helSyn or α-P_4,5_helSyn antibodies were reprobed with α-helSyn antibody after the inactivation of peroxidase activity by its substrate, hydrogen peroxide. This method, unlike classical stripping, avoids the loss of proteins from membranes. As we have verified in several tests, the chemiluminescent signal of the first secondary antibody is completely quenched by treatment with hydrogen peroxide without interfering with following reprobings. As shown in Fig. 6B,C, KCl treatment induced a significant increase in synapsin phosphorylation state at both site 1 (264.6±30.08%, *n* = 7, *P*<0.001 Student’s *t*-test) and sites 4 and 5 (214.2±43.66%, *n* = 5, *P*<0.05 Student’s *t*-test) with respect to control. Interestingly, PTZ is also able to induce a statistically significant, almost two-fold, increase in phosphorylation. In particular, we measured a phospho-ratio of 176.9±18.01% (*n* = 7, *P*<0.01 Student’s *t*-test) for phospho-site 1, and a phospho-ratio of 204.2±40.41% (*n* = 5, *P*<0.05 Student’s *t*-test) for phospho-sites 4 and 5, with respect to control value.

**Figure 7:**
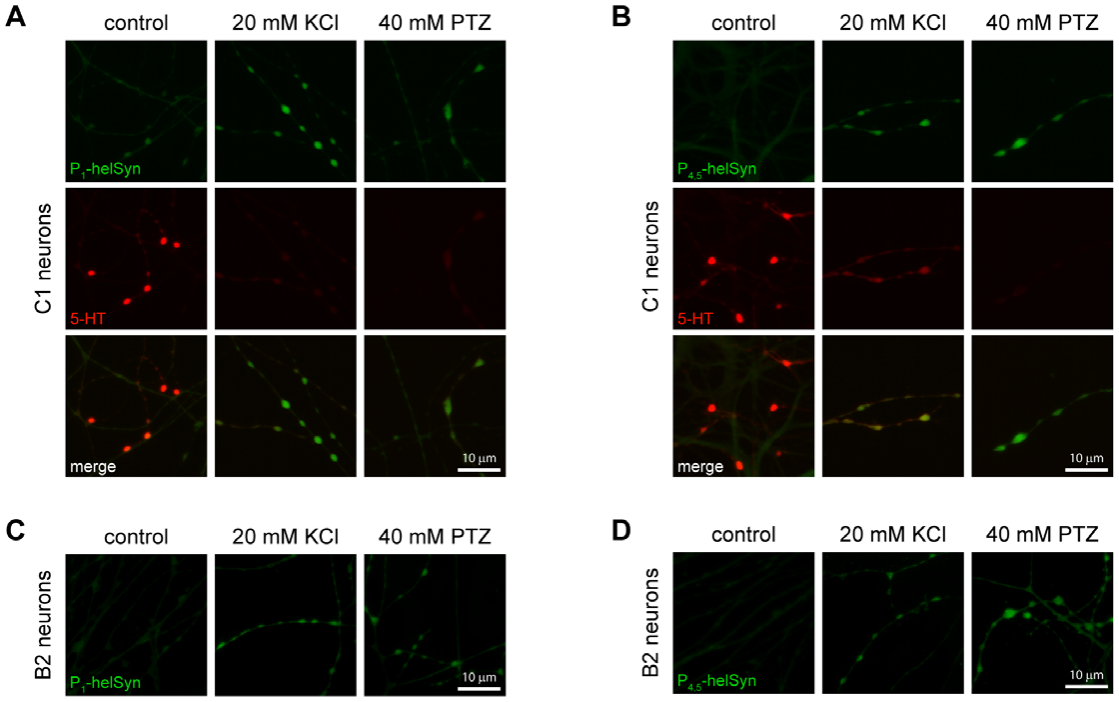
PTZ-induced epileptiform activity promotes massive neurotransmitter release and phosphorylation of synapsin at presynaptic varicosities. Epifluorescence micrographs sample images of *Helix* C1 neurites and varicosities immunolabelled with rabbit α-P_1_helSyn (A) or α-P_4,5_helSyn antibodies (B), in green. Cells were fixed after 15 minutes of incubation with culture medium (control, left of each panel), or 20 mM KCl (middle of each panel) or 40 mM PTZ (right of each panel). Both treatments increased synapsin phospho-levels of site 1 and sites 4/5. A double staining for serotonin (5-HT), in red, revealed a strong loss of immunoreactivity, indicative of a massive neurotransmitter release in KCl and PTZ-treated cells. Sample images of *Helix* B2 neurites and varicosities treated as above described and then immunolabelled with rabbit α-P_1_helSyn (C) or α-P_4,5_helSyn antibodies (D). Scale bars: 10 μm.

Previous studies indicate that synapsin is localised within presynaptic terminals in the regions occupied by synaptic vesicles [28], [54]–[62]. We performed immunostaining of cultured serotonergic *Helix* C1 (Fig. 7A,B) and B2 (Fig.7C,D) neurons to specifically quantify the PTZ-induced synapsin phosphorylation at presynaptic structures, namely varicosities. We observed a basal low-level of fluorescence for both synapsin phospho-sites in untreated cells, while a double staining for serotonin revealed the presence of this neurotransmitter in the same structures (left panels). By contrast, in neurons treated with 20 mM KCl, a several-fold increase in phospho-synapsin immunoreactivity occurred in presynaptic structures, where synapsin is predominantly localised (central panels). A similar enhancement of fluorescence level for site 1, as well for sites 4 and 5, was clearly observable in neurons fixed after the exposure to 40 mM PTZ (right panels). Interestingly, we observed a strong decrease in serotonin immunoreactivity in C1 cells stimulated with either high potassium or PTZ, confirming that both KCl-induced depolarisation and epileptiform activity led to a massive release of neurotransmitter and vesicle depletion.

**Figure 8:**
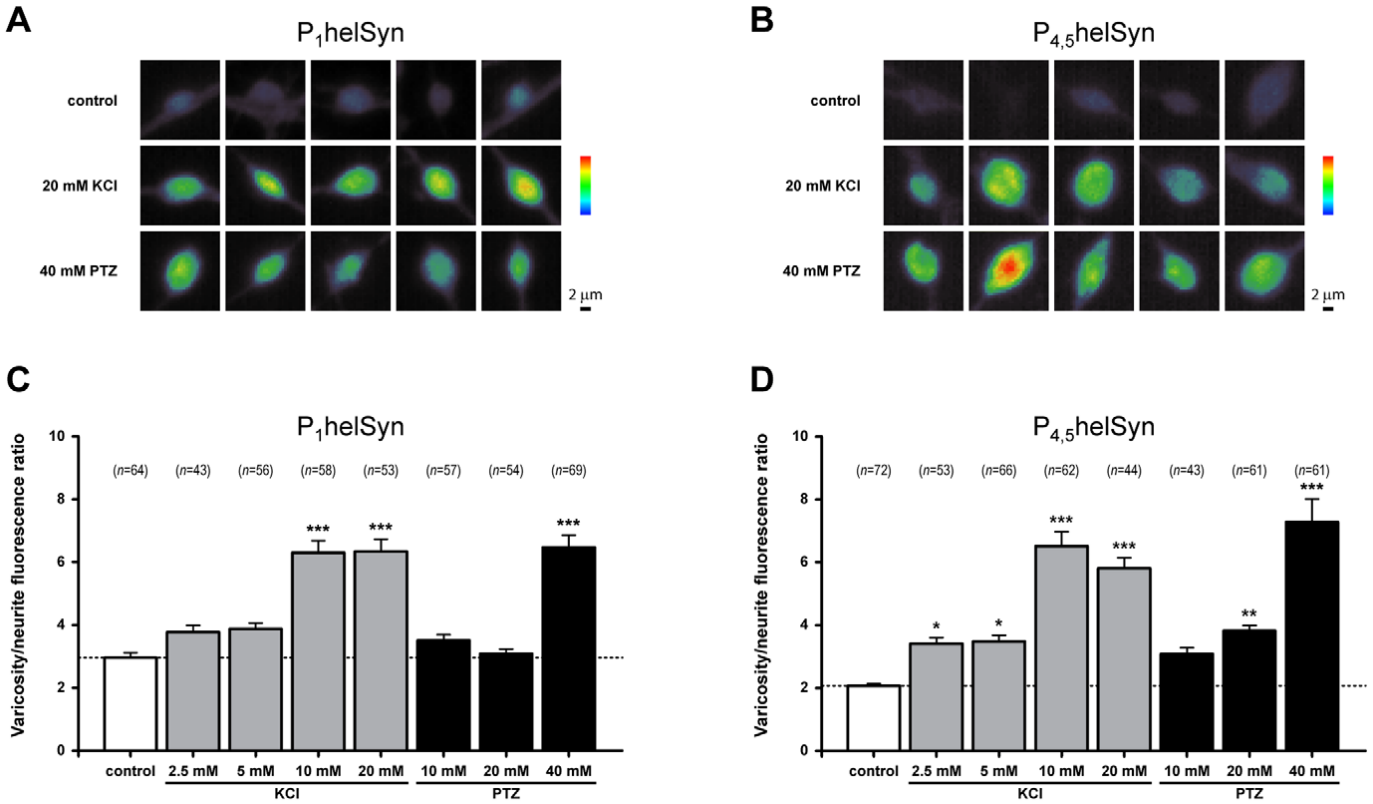
PTZ-application promotes phosphorylation of synapsin domain A and B in a dose-dependent fashion. (A,B) Representative acquisitions of B2 varicosities expressed in pseudocolour, from purple (scarcely stained) via green (weakly stained) to red (most strongly stained), to better appreciate differences in synapsin phosphorylation. Treatments with 20 mM KCL and 40 mM PTZ were shown. Scale bars: 2 μm. (C,D) Quantification of synapsin phospho-levels from varicosities of B2 neurons processed with the indicated treatments and immunolabelled with either α-P_1_helSyn or α-P_4,5_helSyn antibodies. A ratio between the intensities of varicosity and its proximal neurite has been calculated. The corresponding number of examined varicosities (*n*) is indicated on each bar.

To further test the idea that the level of synapsin phosphorylation is enhanced during epileptiform activity, we quantified the fluorescence intensity from varicose structures of *Helix* B2 neurons (Fig. 8A,B) treated with either subthreshold (10 or 20 mM) or epileptogenic concentration (40 mM) of PTZ. As a positive control, cells were stimulated with various concentrations of KCl (2.5, 5, 10 or 20 mM). Variations in phospho-synapsin levels were taken into account by normalizing the relative amounts of fluorescence detected in varicosities with those measured in their proximal neurite. We calculated a varicosity/neurite intensity ratio that also relies on synapsin localisation at presynaptic structures (modified from [63]). As regards helSyn PKA/CaMKI/IV and MAPKs phosphorylation sites, one-way ANOVA revealed a significant effect of treatment for both phospho-site 1 (F_(7,446)_ = 30.46, *P*<0.0001; Fig. 8C) and phospho-sites 4 and 5 (F_(7,454)_ = 27.75, *P*<0.0001; Fig. 8D), respectively.

Considering synapsin phospho-site 1, we found that 10 mM KCl is a sufficient concentration to significantly increase its phosphorylation level (varicosity/neurite ratio: 6.30±0.38 *vs*. 2.95±0.17 of control group; *P*<0.001, Bonferroni’s *post hoc* test). Interestingly, B2 cells treated with subthreshold concentrations of PTZ exhibited similar fluorescence intensities to those measured in untreated neurons (3.51±0.19 and 3.08±0.16 for 10 mM and 20 mM PTZ, respectively). Conversely, the application of 40 mM PTZ resulted in a two-fold increase in varicosity/neurite ratio (6.48±0.39; *P*<0.001, Bonferroni’s *post hoc* test), thus indicating synapsin phospho-site 1 as a molecular target during epileptiform activity.

Immunostaining specific for synapsin phospho-sites 4 and 5 showed an enhanced phosphorylation status directly proportional with increasing concentrations of KCl (varicosity/neurite ratio: control: 2.07±0.07; 2.5 mM KCl: 3.41±0.19, *P*<0.05; 5 mM KCl: 3.48±0.19, *P*<0.05; 10 mM KCl: 6.51±0.46, *P*<0.001; 20 mM KCl: 5.81±0.33, *P*<0.001, Bonferroni’s *post hoc* test). Although 10 mM PTZ was not sufficient to significantly change the phosphorylation level of these sites (3.08±0.20), we found a moderate increase in the varicosity/neurite ratio with the application of 20 mM PTZ (3.82±0.17, *P*<0.01, Bonferroni’s *post hoc* test). Similar to previous results, a 3.5-fold enhancement of this ratio was detected with the epileptogenic concentration of 40 mM (7.28±0.73, *P*<0.001, Bonferroni’s *post hoc* test), indicating that synapsin domain B, as well as domain A, is a target of PTZ-promoted phosphorylation events.

**Figure 9:**
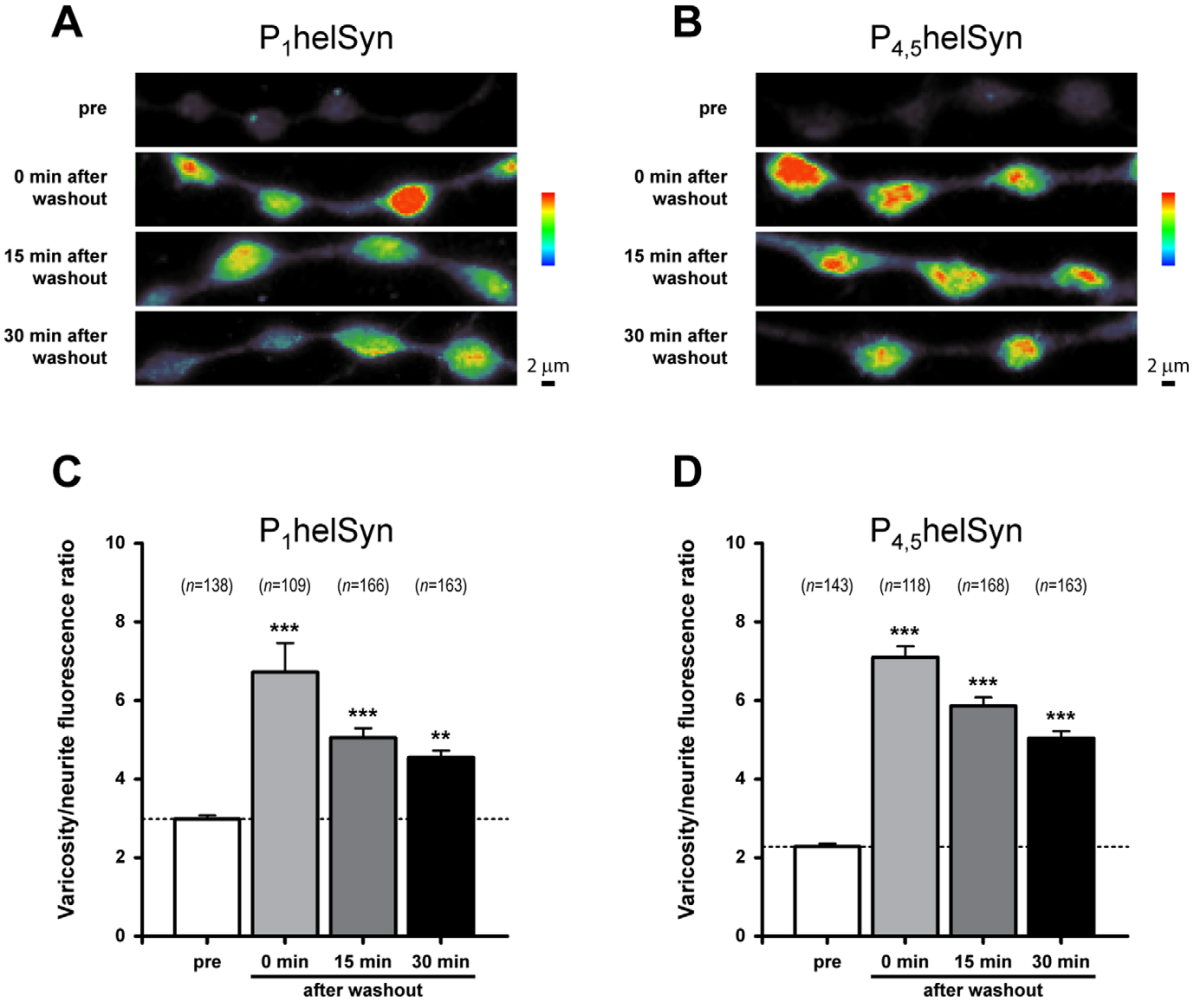
PTZ-induced epileptiform activity promotes a prolonged phosphorylation of synapsin domain A and B. (A,B) Pseudocolour representative acquisitions of B2 varicosities and neurites fixed before 40 mM PTZ treatment and every 15 minutes after drug washout. Cells were then immunolabelled with either α-P_1_helSyn or α-P_4,5_helSyn antibodies, as indicated. Scale bars: 2 μm. (C,D) Bar graphs showing the mean varicosity/neurite fluorescence ratio for synapsin phospho-levels measured as previously reported. A statistically significant increase in synapsin phosphorylation at both phospho-sites was observed even 30 minutes after PTZ washout. The corresponding number of examined varicosities (*n*) is indicated on each bar.

In another set of experiments, we quantitatively determined the phosphorylation state of synapsin measuring the varicosity/neurite ratio in cells treated with 40 mM PTZ for 15 minutes and then fixed soon after washout, or 15 and 30 minutes after drug removal (Fig. 9A,B). We observed that PTZ treatment promoted a remarkable enhancement of varicosity/neurite ratio in phospho-site 1, (F_(3,572)_ = 18.83, *P*<0.0001, one-way ANOVA; Fig. 9C) from 2.99±0.09 (pre-treatment) to 6.72±0.74 (0 min after washout; *P*<0.001, Bonferroni’s *post hoc* test). At 15 and 30 minutes after washout we determined a varicosity/neurite ratio of 5.06±0.24 (*P*<0.001, Bonferroni’s *post hoc* test) and 4.55±0.17 (*P*<0.01, Bonferroni’s *post hoc* test), respectively. Therefore, the increased phosphorylation level of synapsin in site 1 appeared to show a slow declining tendency after PTZ removal, remaining significantly higher with respect to control even 30 minutes after epileptiform activity.

Similar results were observed for phospho-sites 4 and 5 where drug-induced epileptiform activity promoted a statistically significant increase in varicosity/neurite ratio (F_(3,588)_ = 102.4, *P*<0.0001, one-way ANOVA; Fig. 9D) from 2.28±0.08 (pre-treatment) to 7.10±0.27 (0 min after washout; *P*<0.001, Bonferroni’s *post hoc* test). Lower values, but still significantly higher with respect to control, were detected at 15 and 30 minutes after washout, 5.86±0.23 (*P*<0.001, Bonferroni’s *post hoc* test) and 5.05±0.17 (*P*<0.001, Bonferroni’s *post hoc* test), respectively.

In conclusion, the data obtained from western blot assay and immunostaining analysis provide strong evidence that the PTZ-induced epileptiform activity leads to an increase in synapsin phosphorylation level at both phospho-sites 1 and 4/5, possibly through the activation of the corresponding kinase pathways, i. e. PKA, CaMKI/IV and MAPK/Erk.

## Discussion

### Epileptiform activity alters basal synaptic transmission

Reliable synaptic transmission is supported by proper organisation of synaptic vesicle pools. Neurotransmission failure may result from sustained neural activity, such as during an epileptiform episode, where continuous exocytosis of neurotransmitter prevails over mechanisms that replenish neurotransmitter availability. Indeed, epileptic seizures induce remarkable alterations of the dynamics of vesicle redistribution into the synaptic apparatus. Ultrastructural studies on synapses of the rat central nervous system revealed two opposing changes after seizure termination: an immediate dramatic depletion of synaptic vesicles [64], similar to that observed after other intense stimulation conditions [65], [66], and a successive increase in the number of vesicles near the active zone [67], [68]. This relocation of vesicles towards the synaptic cleft contributes to raising the activity of the epileptic focus area, increasing the supply of neurotransmitter [69]. Although synaptic fatigue during epileptic-like activity may be protective against excessive excitability in the nervous system, the strengthening of excitatory synapses may worsen the epileptic phenotype.

Our results corroborate this evidence. The observed gradual decline of EPSP amplitude and the marked reduction in serotonin immunoreactivity during epileptiform activity confirm the loss of neurotransmitter from presynaptic structures. By using a rapid depletion technique [49], we have also estimated that the size of the RRP is reduced soon after drug washout without changes in the vesicle replenishment rate. However, no alteration in basal transmission is detectable at this time-point (0 min after washout), since similar values in EPSP amplitude and time course were recorded before and after treatment. This discrepancy could be explained with the parallel strong increase in P_rel_, deduced from the cumulative EPSP plots, that may compensate for the lack of vesicles during basal stimulation.

Previous studies have shown that the paired-pulse ratio is inversely related to release probability, and has been frequently used to examine vesicular release dynamics [51]–[53]. At *Aplysia* synapses, changes in PPR are predominantly determined by P_rel_ [70], [71]. This analysis revealed a transient reduction in PPR at B2–B2 synapses following PTZ treatment, corresponding to an increase in P_rel_, consistent with our interpretation of cumulative EPSP plots. Hence, these data suggest a dynamic modulation of P_rel_ after epileptiform activity that may affect release kinetics, since a concomitant shortening of EPSP rise times has been observed. Both parameters completely recovered at 30 minutes after drug removal. In our model, this increase in probability and speed of neurotransmitter release may reflect a positive modulation of release machinery induced by epileptiform activity, perhaps through transient protein modification such as phosphorylation or increased Ca^2+^ concentration in the presynaptic terminals [72]. Alternatively, we cannot exclude the rapid activation of other active zones that were previously silent [73]. An enhancement in amplitude of evoked postsynaptic currents and a shortening of their rise times have also been described in basolateral amygdala neurons of rats which undergo to epileptic discharges induced by electrical kindling [74]. Shoji and colleagues proposed that these modifications probably resulted from an increase in P_rel_ at nerve terminals. Indeed, our evidence supports these findings.

We also observed that basal transmission not only recovers at 15 and 30 minutes post-PTZ treatment, but also shows a significant increase in EPSP amplitude which rapidly decays to control values following a few action potentials. This decay exhibits a time course comparable to that described in homosynaptic depression of other gastropod synapses [75]–[77]. Here, these differences in synaptic efficacy may be ascribed to opposite changes between the size of the RRP and the P_rel_. While the higher P_rel_ required 30 minutes after drug washout to return to pre-treatment level, the estimated RRP showed a progressive increase in size. Imbalances of these two parameters have often been associated with facilitating and depressing synapses [78]. In previous studies on *Helix* neurons, some forms of synaptic enhancement have been attributed to postsynaptic receptor sensitisation [79]. By applying small amounts of serotonin, we assessed that the overall neurotransmitter responsiveness of treated B2 neurons does not change after PTZ exposure at different time-points. Nevertheless, we cannot totally exclude a possible interaction because it is difficult to discriminate between extrasynaptic receptors and postsynaptic receptors in the synaptic cleft which may show different functional properties depending on their localisation [80]–[82].

Taking into account that we never observed synaptic depression at B2–B2 synapses *in vitro*, we define the above described EPSP amplitude decrease as a depotentiation rather than a synaptic depression, since values decline to a baseline that is similar to the pre-treatment mean EPSP amplitude. In view of this, we can speculate that chemically-induced epileptiform activity effectively promotes a short-lasting form of synaptic plasticity in our system. Our observations bear resemblance to a novel form of short-term potentiation described in reconstructed synapses between *Lymnaea* VD4 and LPeD1 neurons, which exhibit an enhancement of basal transmission with comparable decay kinetics following tetanisation [83]. In both cases, there is an increase in the first EPSP amplitude followed by a rapid depotentiation upon triggering a few action potentials. The same synaptic enhancement is displayed when stimulated at different time-points, since we observed that the potentiated response is similar from 15 to 30 minutes following epileptiform activity. On the basis of these properties, these forms of plasticity have been defined as use- rather than time-dependent [83]. A similar use-dependent dynamic upregulation of synaptic transmission has also previously been described in the CA1 area of rat hippocampus [84], thus excluding that these synaptic modulations are restricted to invertebrate neurons.

The precise mechanisms by which use-dependent plasticity is elicited still remain unknown. However, new evidence has emerged to support a direct involvement of the same signal transduction pathways already implicated in modulation of neurotransmitter release. For instance, use-dependent potentiation at *Lymnaea* VD4–LPeD1 synapse requires the activation of Ca^2+^calmodulin dependent kinase II (CaMKII) in presynaptic terminals. As a downstream target, synapsin is one of the putative phosphorylation substrates that has been proposed to play a role in this form of plasticity [83].

### Epileptiform activity alters post-tetanic potentiation

Post-tetanic potentiation is a form of short-term homosynaptic plasticity triggered by high-frequency stimulation. The resulting increase in synaptic efficacy is a sum of several concurrent mechanisms that modulate the efficacy of neurotransmitter release [85]–[87] and the different steps of the synaptic vesicle cycle [85], [86], [88], [89]. While it has been clearly demonstrated that PTP strongly correlates with an increase in the RRP, due to the recruitment of synaptic vesicles from the RP during high-frequency activity [32], [90], [91], the degree of contribution of P_rel_ to PTP maintenance is still debated. It has been demonstrated that the tetanus-induced enhancement of P_rel_ decays rapidly with a fast time constant not comparable with PTP decay kinetics [92].

In the present work, we show that PTP is significantly affected in B2–B2 monosynaptic connections following epileptiform activity, with a large decrease in both amplitude and duration that lasts even 30 minutes after PTZ removal. Since 15 minutes is a sufficient period of time to allow the complete re-establishment of synaptic functionality (see control group), we can therefore speculate a PTZ-induced alteration of the molecular effectors responsible for PTP expression and maintenance. One possible explanation of the PTP deficit is a partial occlusion resulting from prolonged firing activity, however this hypothesis is in contrast with our results. Following PTZ application, at B2–B2 synapses we observed an unaltered vesicle replenishment rate, similar postsynaptic responses to neurotransmitter application, an enhancement of P_rel_ and faster kinetics of vesicle release, deduced from the shorter rise time of EPSPs. Taking into account these elements, PTP would be expected to increase in its amplitude and/or decay rather than being impaired.

To conclude, our experiments on basal transmission and PTP expression may suggest an impairment in dynamic reorganisation of synaptic vesicle pools following the massive release of neurotransmitter due to the sustained firing induced by PTZ. The supply of synaptic vesicles from the RP to the RRP is one of the key steps that sustain the enhanced neurotransmitter release during PTP [33], [85], [91]–[93]. As previously mentioned, synapsin is a good candidate for such dysregulation in the presynaptic terminal, since it is clearly implicated in maintaining presynaptic vesicular pools and in regulating vesicle mobility during short-term plasticity [94]–[98].

There are several lines of evidence suggesting a key role of synapsin in PTP both in mammalian and invertebrate synapses. Mice lacking synapsin II or double synapsin I/II knockouts exhibit a strong reduction in PTP and learning deficits [16], [99]. Intraneuronal injection of specific anti-synapsin antibodies reduces PTP at *Aplysia* cholinergic synapses [94]. In *Helix* neurons we previously demonstrated that PTP is strongly related to PKA, CAMKI/IV and MAPK/Erk kinase pathway activation and thus to the phosphorylation of helSyn at the corresponding consensus sites [43], [44]. In agreement with our hypothesis, the PTZ-induced reduction in PTP amplitude observed in this study is similar to those described in B2–B2 synapses overexpressing a pseudophosphorylated mutant form of helSyn presynaptically [44]. These data suggest that cycles of phosphorylation/dephosphorylation are required for proper activity of synapsin during synaptic plasticity. The fact that we observed a prolonged phosphorylation state in both synapsin phospho-sites (high levels still present 30 minutes after PTZ washout) may explain the persistence of PTP impairment at this time-point. Recently, the importance of these phosphorylation sites for PTP expression has also been confirmed in mouse glutamatergic autapses [100].

### Epileptiform activity induces synapsin phosphorylation

Synapsins have been frequently implicated in epilepsy. The single, as well as the double and triple, synapsin knockout mice suffer from epileptic seizures [15], [16], [18], [19], [21], consistent with an imbalance in excitability of the nervous system. Genetic screens identify several mutations in synapsin genes associated with human epilepsy and autism disorders [17], [20]. Moreover, biochemical studies have indicated that administration of convulsant drugs, i.e. PTZ or picrotoxin, results in an increased phosphorylation level of a wide range of intracellular proteins [101], including synapsin I [102]. Although an extensive identification of these substrates still remains to be achieved, the phosphorylation events associated with PTZ application seem to be calcium-dependent since they are reduced in calcium-free medium or in presence of chelating agents [103]–[106]. Hence, a wide range of kinase pathways and molecular effectors are most likely activated during PTZ-induced epileptiform activity and therefore may contribute to the overall effects in synaptic functions that we described in our experimental model.

**Figure 10:**
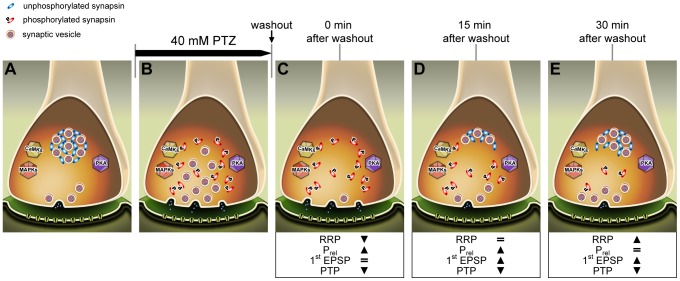
Epileptiform activity increases the synapsin phosphorylation status, thus leading to an altered basal synaptic transmission. Hypothetical cascade of intracellular events occurring at presynaptic terminal during epileptiform activity. At resting potential (A), unphosphorylated synapsin tethers small vesicles into the RP. Prolonged paroxysmal activity (B) that induces the activation of several kinase pathways and promotes vesicle mobilization through synapsin phosphorylation, leads to a very large release of neurotransmitter (C). Afterwards, although recycled vesicles start to reorganize themselves into synaptic pools (D), the enhanced phosphorylation status of synapsin compromises pool maintenance, thus increasing the number of free vesicles that are ready to be released (E).

In this work, we have selectively analysed the phosphorylation levels of *Helix* synapsin domain A (site 1) and B (sites 4 and 5) regulated by PKA/CaMKI/IV and MAPK/Erk, respectively. Our findings show that synapsin phosphorylation is strongly enhanced in both sites by application of PTZ at the epileptogenic dose of 40 mM, but not at 20 mM, defined as a subthreshold concentration [9], [48]. Interestingly, intracellular recordings reveal that application of either 20 or 40 mM PTZ triggers a similar mean firing rate (about 1 Hz), but only the concentration of 40 mM determines the switch to burst activity and PDS. Therefore, we may speculate that synapsin phosphorylation is directly promoted by epileptiform activity rather than by the prolonged low frequency firing. A large body of literature suggests that phosphorylation of synapsin site 1 results in decreased actin [107] and synaptic vesicle binding [23], while sites 4 and 5 seem to be predominantly involved in triggering actin polymerisation and bundling [108]. Through this mechanism, both phospho-sites play an important role in modulating neurotransmitter release in vertebrates [39], [109]–[112] and invertebrates [43], [44], [113]. According to our results, a prolonged phosphorylation of synapsin may be responsible for both the increase in the RRP size, thus altering basal synaptic transmission, and the alteration of vesicle pool organisation (e.g. clustering vesicles into the RP), leading to PTP impairment. It is likely that, after vesicle depletion, recycled vesicles might be clustered less efficiently into the RP, thus increasing the number of releasable vesicles (see Fig. 10).

The pathologic activation of the same molecular pathways that regulate synaptic strength in short- and long-term plasticity may lead to an imbalance between excitatory and inhibitory transmission degenerating into an epileptic episode. Accordingly, evidence from several animal models suggests a possible role for both PKA [114]–[119] and MAPK/Erk [120]–[124] in the development and maintenance of increased neural excitability and epileptiform activity.

## Conclusions

In this work, we provide compelling evidence that prolonged epileptiform activity leads to an increase in the synapsin phosphorylation state, possibly losing synaptic vesicle pool segregation, thus contributing to an alteration of synaptic strength in both the basal condition and tetanus-induced potentiation. Through this mechanism, the long-lasting firing activity and the repetitive phosphorylation events may induce a protracted modification of synaptic transmission which could dramatically increase the severity of epileptic seizures.

## Methods

### Materials

All reagents and materials were of analytical grade and purchased from Sigma (Milan, Italy), unless stated otherwise.

### Cell culture

Juvenile *Helix aspersa* land snails were purchased from local breeders. Cell cultures were performed as previously described [14], [44], under either adhesive or non-adhesive conditions.

### PTZ treatment

In order to induce epileptiform activity in *Helix* neurons, PTZ was dissolved in L-15 Leibovitz culture medium to a final concentration of 40 mM (epileptogenic dose; see [9], [48]). Where expressively specified, subthreshold concentrations of 10 and 20 mM were employed. In some experiments, isolated or chemically interconnected B2 neurons were perfused with PTZ solution during electrophysiological recording. After 15 minutes, PTZ was rapidly washed out (7.5 ml/min) with 5 volumes of the recording chamber (about 10 ml). Flow was actively controlled by a peristaltic pump (Ismatec ISM829; Glattbrugg, Switzerland). Cells that exhibited neither bursting activity nor PDS were not included. As a control, the same stimulation protocols were applied with the perfusion of culture medium for 15 minutes without the presence of PTZ (control).

### Electrophysiological recording

Conventional intracellular recordings of synaptic activity were performed in current-clamp mode as previously reported [43], [44]. Signals were recorded with an Axoclamp 900A amplifier (Molecular Devices, Sunnyvale, CA, USA) and digitally recorded via a Digidata 1322A interface (Molecular Devices). Offline analysis was performed with Axoscope and Clampfit software products (Molecular Devices).

The response to focal application of serotonin was evaluated before and after PTZ treatment on B2 neurons kept hyperpolarised 30 mV below their resting potential (about -80 mV) to prevent firing. A solution of 20 µM serotonin in L-15 medium was loaded into a patch-clamp glass electrode and focally applied using short pressure pulses (5 pulses of 1 second at 10 psi) delivered through a pneumatic picopump (PV820, WPI, Sarasota, FL), connected to the electrode holder. In each experiment, the tip of the electrode was positioned 50 m distance from the somata, corresponding to a representative B2 cell diameter. Measured postsynaptic responses were averaged to provide the amount of depolarisation induced by each pulse. In order to avoid the phenomenon of desensitisation, neurons were adequately perfused with fresh medium between each application.

To analyse epileptiform-induced changes in basal synaptic transmission and short-term plasticity, B2–B2 pairs were tested 48 hours after cell-cell pairing for the presence of a chemical monosynaptic connection. Soma-soma pairs that showed electrical coupling were not used. Generally, presynaptic neurons were stimulated five times to fire action potentials and corresponding EPSPs were recorded in postsynaptic cells kept hyperpolarised to -80 mV. Suprathreshold stimulation was triggered by a Pulsemaster A300 stimulator (WPI, Sarasota, FL, USA) at the basal frequency of 0.05 Hz before (pre-treatment) and after perfusion of a 40 mM PTZ solution for 15 minutes, starting 0 min after washout. Afterwards, synapses were tested again at 15 and 30 minutes after drug washout. During the entire protocol, neurons were continuously monitored to check any spontaneous activity between stimulations. To evaluate the time course of EPSP amplitude, values were expressed as percentage of the first evoked EPSP. EPSP rise times were measured between 10% and 90% of its peak amplitude.

The induction and quantification of PTP at B2–B2 synapses were performed as previously reported [44]. For statistical analysis, post-tetanic EPSP amplitudes were normalised to the mean amplitude of the last five pre-tetanic EPSPs. The PTP decay time constant τ was obtained by exponential curve-fitting procedures using the statistical software GraphPad Prism ver.5 (GraphPad Software, San Diego, CA, USA).

### Estimation of the RRP size, P_rel_ and PPR

The size of the RRP and P_rel_ were calculated at different time-points in both PTZ-treated and untreated monosynaptic connections by performing a cumulative amplitude analysis [49]. Following this method, we measured the cumulative sum of EPSP amplitudes during 100 repetitive stimuli applied at 10 Hz (depleting stimulus). During stimulation, EPSPs declined and reached a steady-state, typically interpreted as the ongoing replenishment of the depleted RRP [49], [125], [126]. The number of data points to include in the linear fitting of the steady-state phase was evaluated by calculating the best coefficient of determination (R^2^) comprising the maximal number of values starting from the last stimulus. At B2–B2 synapses, the cumulative amplitude profile showed the best linear course after the first 25 stimuli, in both treated and untreated synapses. Thus, all data points in the range between 25 and 100 were fitted by linear regression obtaining R^2^ values >0.99 at each examined synapse. The *y*-intercept of fitted line, back-extrapolated at time 0, was used as an estimate of the number of quanta in the RRP, expressed in mV. The slope of the regression line provided an indicator of the rate of vesicle replenishment.Subsequently, the probability of release (P_rel_) was calculated as the ratio between the first EPSP evoked in the train and the estimated RRP size.

For each train of EPSPs, the paired-pulse ratio (PPR) was calculated as the ratio between the amplitude of the second EPSP and that of the first one (i.e. EPSP2/EPSP1 amplitude) with an interpulse interval of 100 ms.

### Phospho-specific helSyn antibodies and dephosphorylation assay

For *Helix* Synapsin (helSyn), the phospho-peptides CLRRRF(S9)*SGDLQGEANEKED (PKA and CaMKI/IV phosphorylation site on Ser 9, indicated as site 1), CNFKKGP(S36*)PS and APN(S42*)PSKSAC (MAPK/Erk phosphorylation sites on Ser 36 and 42, respectively named sites 4 and 5) were manufactured by Inbios International Inc. (WA, USA). OVA or SMCC-conjugated peptides were used to immunise rabbits and the resultant sera were affinity-purified (antibody anti-helSyn phosphorylated on site 1, α-P_1_helSyn) or supplied unpurified (antibody anti-helSyn phosphorylated on site 4 and 5, α-P_4,5_helSyn) (Inbios International Inc.). The specificity of helSyn phospho-specific antibodies was tested on western blot by means of a dephosphorylation assay. Specifically, a western blot was performed, in which the same sample was loaded several times. The membrane was divided into four parts. Two parts, intended as controls, were probed with either α-helSynP_4,5_ or α-helSynP_1_ according to the protocol described in the following section. The other two parts underwent a treatment with Calf-intestinal alkaline phosphatase (CIAP, 1 U/ml, in presence of its buffer) (Promega, WI, USA), that catalyzes the removal of phosphate groups from the blotted proteins, overnight at 37°C with gentle shaking. The day after, these membranes were rinsed twice in TTBS and probed with the phospho-specific antibodies. No bands appeared on the films thus confirming the specificity of helSyn phospho-specific antibodies. To exclude protein loss, the same membranes were re-probed with α-helSyn antibody (Inbios International Inc.), which recognizes *Helix* synapsin, either phosphorylated or not.

### Western blot

For 7 independent experiments, cerebral ganglia and the subesophageal ring were dissected from adult specimens of *Helix aspersa*. Ganglia were transferred in L-15 medium alone (control) or with addition of 40 mM PTZ for 1 hour or 60 mM KCl for 5 minutes. Next, ganglia were quickly frozen in liquid nitrogen and stored at -80°C until lysis. Homogenates were prepared in a Mixer Mill MM 400 apparatus (Retsch, Verder Group, Netherlands) in 20 mM TrisHCl, pH 7.5, containing 1% SDS and protease inhibitors (1 mM Sodium orthovanadate, 2 mM Sodium fluoride, 2 mM Sodium pyrophosphate, 1 µg/ml Aprotinin, 1 µM Pepstatin A, 5 mM EDTA, 1 mM DTT, 1 mM PMSF). Aliquots of 35 µg total proteins were separated on a 5% to 8% SDS-polyacrylamide gel and transferred onto a nitrocellulose membrane (Amersham Hybond, GE Healthcare, UK) before being blocked for 1 hour at room temperature (RT) and then probed overnight at 4°C with primary antibody diluted to appropriate concentrations (α-helSyn 1∶700; α-P_1_helSyn 1∶150; α-P_4,5_helSyn 1∶100; polyclonal rabbit α-actin 1∶200, Sigma). Blots were then incubated with a horseradish peroxidase-conjugated goat α-rabbit IgG antibody (1∶800, Thermo Fisher Scientific, MA, USA) for 1 hour at RT and visualised using SuperSignal WestPico Chemiluminescent reagent (Thermo Fisher Scientific). Hybridisation with phospho-specific antibodies were subjected to reprobing with α-helSyn after the inactivation of peroxidase activity by hydrogen peroxide [127], [128]. Western blot films were scanned and the adjusted volume (after background subtraction) of bands was quantified using Quantity One software (Bio-Rad Laboratories, CA, USA). Blots probed with α-helSyn were normalised with actin. We defined the phospho-ratio parameter as the ratio between the phospho-specific antibodies and α-helSyn antibody immunoreactivity. The effects of KCl and PTZ were calculated as the fold change in phospho-ratio between control and treated samples.

### Immunocytochemistry and fluorescence analysis

To analyse the phosphorylation level of helSyn in varicose structures, B2 neurons and serotonergic C1 neurons were isolated and cultured for 3 days under adhesive conditions to promote neurite growth. After treatment for 15 minutes with appropriate concentrations of PTZ or KCl, cells were rapidly fixed with 4% paraformaldehyde in 0.1 M phosphate-buffered saline (PBS; 45 minutes at RT). Where indicated, treated cells were washed with L-15 culture medium and fixed after a resting period of 15 or 30 minutes. Cultures were then rinsed with PBS (3 times; 10 minutes), blocked with 5% bovine serum albumin and PBS plus 0.25% saponin (PBS^+^) (1 hour at RT). Permeabilised cells were subsequently incubated overnight at 4°C with suitable primary antibodies appropriately diluted in blocking buffer. After washing with PBS^+^, cultures were incubated in blocking buffer with fluorescent labelled secondary antibodies. After 1 hour at RT, neurons were rinsed with PBS^+^ and examined using an Eclipse TE200 inverted microscope (Nikon Instruments, Tokyo, Japan) equipped with phase contrast and epifluorescence optics. Images were captured with a Monochrome Evolution QE camera (MediaCybernetics, Bethesda, MD, USA). The following antibodies were used: rabbit anti-P_1_-helSyn (1∶250) and anti-P_4,5_-helSyn (1∶250) custom-designed polyclonal antibodies (Inbios International Inc.); mouse anti-serotonin monoclonal antibody (1∶70; Dako A/S, Denmark); rhodamine-conjugated goat anti-mouse (1∶200; Sigma); fluorescein-conjugated goat anti-rabbit (1∶200; Sigma).

Quantitative analysis of phospho-synapsin fluorescence were performed using the Line Profile tool of Image Pro Plus version 6.3 (MediaCybernetics). A varicosity/neurite ratio was calculated from background-subtracted intensity values measured from varicosities and their proximal neurites. This parameter takes into account the synapsin localisation at presynaptic terminals [63]. Small varicosities with a size less than 1.5 µm (corresponding to about 2 µm^2^ of area) were not included in analysis. The amount of phosphorylation was referred to the ratio obtained from untreated cells.

All figures were assembled using Photoshop CS2 version 9.0 software (Adobe Systems, San Jose, CA).

### Statistical analysis

Data were expressed as means ± s.e.m. Statistical analysis was performed using GraphPad Prism version 5 (GraphPad Software). The statistical significance between group means was assessed using Student’s *t*-test or ANOVA analysis (one or two-way and with or without repeated measures where appropriate) followed by Bonferroni’s *post-hoc* test. Significance levels were set at *P*<0.05. In the figures, asterisks indicate following significance levels *vs*. respective control group: ***, *P*<0.001; **, *P*<0.01; *, *P*<0.05.
